# The Response to Oxidative Damage Correlates with Driver Mutations and Clinical Outcome in Patients with Myelofibrosis

**DOI:** 10.3390/antiox11010113

**Published:** 2022-01-05

**Authors:** Elena Genovese, Margherita Mirabile, Sebastiano Rontauroli, Stefano Sartini, Sebastian Fantini, Lara Tavernari, Monica Maccaferri, Paola Guglielmelli, Elisa Bianchi, Sandra Parenti, Chiara Carretta, Selene Mallia, Sara Castellano, Corrado Colasante, Manjola Balliu, Niccolò Bartalucci, Raffaele Palmieri, Tiziana Ottone, Barbara Mora, Leonardo Potenza, Francesco Passamonti, Maria Teresa Voso, Mario Luppi, Alessandro Maria Vannucchi, Enrico Tagliafico, Rossella Manfredini, on behalf of the Mynerva (MYeloid NEoplasms Research Venture AIRC)

**Affiliations:** 1 Centre for Regenerative Medicine, Life Sciences Department, University of Modena and Reggio Emilia, 41125 Modena, Italy; elena.genovese@unimore.it (E.G.); margherita.mirabile@unimore.it (M.M.); sebastiano.rontauroli@unimore.it (S.R.); stefano.sartini@unimore.it (S.S.); sebastian.fantini@unimore.it (S.F.); lara.tavernari@unimore.it (L.T.); elisa.bianchi@unimore.it (E.B.); sandra.parenti@unimore.it (S.P.); chiara.carretta@unimore.it (C.C.); selene.mallia@unimore.it (S.M.); 2 Department of Laboratory Medicine and Pathology, Diagnostic Hematology and Clinical Genomics, AUSL/AOU Policlinico, 41124 Modena, Italy; maccaferri.monica@policlinico.mo.it; 3 Center of Research and Innovation of Myeloproliferative Neoplasms (CRIMM), Department of Experimental and Clinical Medicine, University of Florence, Careggi University Hospital, 50134 Florence, Italy; paola.guglielmelli@unifi.it (P.G.); manjola.balliu@unifi.it (M.B.); niccolo.bartalucci@unifi.it (N.B.); a.vannucchi@unifi.it (A.M.V.); 4 Center for Genome Research, University of Modena and Reggio Emilia, 41125 Modena, Italy; sara.castellano@unimore.it (S.C.); enrico.tagliafico@unimore.it (E.T.); 5 Department of Medical and Surgical Sciences, University of Modena and Reggio Emilia, AUSL/AOU Policlinico, 41124 Modena, Italy; 248358@studenti.unimore.it (C.C.); leonardo.potenza@unimore.it (L.P.); mario.luppi@unimore.it (M.L.); 6 PhD Program in Clinical and Experimental Medicine, University of Modena and Reggio Emilia, 41124 Modena, Italy; 7 Department of Biomedicine and Prevention, University of Tor Vergata, 00133 Rome, Italy; raffaele.f.palmieri@gmail.com (R.P.); tiziana.ottone@uniroma2.it (T.O.); voso@med.uniroma2.it (M.T.V.); 8 Santa Lucia Foundation, I.R.C.C.S., Neuro-Oncohematology, 00179 Rome, Italy; 9 Division of Hematology, Ospedale ASST Sette Laghi, University of Insubria, 21110 Varese, Italy; barbara.mora@asst-settelaghi.it (B.M.); francesco.passamonti@asst-settelaghi.it (F.P.)

**Keywords:** myelofibrosis, calreticulin, janus kinase 2, oxidative stress, reactive oxygen species, superoxide dismutase, 8-hydroxy-2′-deoxy-guanosine, total antioxidant capacity

## Abstract

Myelofibrosis (MF) is the Philadelphia-negative myeloproliferative neoplasm characterized by the worst prognosis and no response to conventional therapy. Driver mutations in *JAK2* and *CALR* impact on JAK-STAT pathway activation but also on the production of reactive oxygen species (ROS). ROS play a pivotal role in inflammation-induced oxidative damage to cellular components including DNA, therefore leading to greater genomic instability and promoting cell transformation. In order to unveil the role of driver mutations in oxidative stress, we assessed ROS levels in CD34+ hematopoietic stem/progenitor cells of MF patients. Our results demonstrated that ROS production in CD34+ cells from *CALR*-mutated MF patients is far greater compared with patients harboring *JAK2* mutation, and this leads to increased oxidative DNA damage. Moreover, *CALR*-mutant cells show less superoxide dismutase (SOD) antioxidant activity than *JAK2*-mutated ones. Here, we show that high plasma levels of total antioxidant capacity (TAC) correlate with detrimental clinical features, such as high levels of lactate dehydrogenase (LDH) and circulating CD34+ cells. Moreover, in *JAK2*-mutated patients, high plasma level of TAC is also associated with a poor overall survival (OS), and multivariate analysis demonstrated that high TAC classification is an independent prognostic factor allowing the identification of patients with inferior OS in both DIPSS lowest and highest categories. Altogether, our data suggest that a different capability to respond to oxidative stress can be one of the mechanisms underlying disease progression of myelofibrosis.

## 1. Introduction

The maintenance of physiological balance between pro-oxidant and antioxidant factors is crucial for proper cellular functions, and the loss of this equilibrium causes oxidative stress. Oxidative stress in turn induces the pathologic accumulation of reactive oxygen species (ROS) that exert their damaging effect on membrane lipids, proteins and DNA [1]. For these reasons, oxidative stress has been investigated in a broad variety of tumors and hematological malignancies, including myeloproliferative neoplasms (MPNs) [2,3].

MPNs are clonal hematopoietic stem cells disorders characterized by excessive production of terminally differentiated myeloid cells [4]. MPNs include three main entities: polycythemia vera (PV), essential thrombocythemia (ET) and primary myelofibrosis (PMF). In particular, PMF patients show high serum level of pro-inflammatory cytokines accompanied by a high level of intracellular ROS, which play a crucial role in chronic inflammation and genomic instability [5]. Moreover, PV and ET can evolve into secondary myelofibrosis (SMF) giving rise to post-PV myelofibrosis (PPV-MF) and post-ET myelofibrosis (PET-MF) [6].

The molecular pathogenesis of PMF has been widely studied in the last years. *JAK2*, *CALR* and *MPL* variants are considered driver mutations since their acquisition plays a key role in leading the malignant clonal expansion [7]. Moreover, these mutations are associated with other DNA alterations which contribute to the onset of the inflammatory state characterized by an excessive production of ROS [2,8]. Several findings support the pathogenetic model in which the oxidative stress contributes to the chronic inflammation and genomic instability [9]. According to this model, an excess of ROS produced by the malignant clone triggers a vicious self-perpetuating circle, in which ROS activate proinflammatory pathways that in turn create more ROS [10,11].

The *JAK2V617F* mutation results in a ligand-independent activation of JAK2 kinase and downstream phosphorylation of STATs [12,13]. It has been demonstrated that *JAK2* mutation leads to an increase of ROS levels through various mechanisms, including p47phox phosphorylation [14] and constitutive activation of AKT/mTOR signaling pathway [15]. Conversely, the inhibition of ROS production could prevent the development of MPNs in a *JAK2V617F* knock-in mouse model, thus confirming the involvement of oxidative stress in the pathogenesis of these myeloid malignancies [16].

In 2013, two independent groups reported the discovery of mutations in *CALR* gene in 60–80% of *JAK2* and *MPL* unmuted ET and PMF patients [17,18]. CALR is a major chaperone in the endoplasmic reticulum (ER) playing multiple roles in several cellular processes, such as quality control of protein folding and calcium homeostasis [19]. Mutations in *CALR* gene mainly consist of insertions or deletions that induce +1-base pair frameshift resulting in a partial loss of the C-terminal domain. It has been shown that physical interaction of mutant CALR homomultimers with MPL causes its constitutive activation [20,21,22]. Wild-type CALR is involved in oxidative stress response in different cell types [23]. Moreover, CALR overexpression has been shown to increase cell sensitivity to H_2_O_2_-induced cytotoxicity [24], indicating that CALR plays a critical role in oxidative stress-induced apoptosis.

We recently demonstrated that K562 cells overexpressing mutated *CALR* display increased sensitivity to oxidative stress, leading to augmented oxidative DNA damage [25]. In order to confirm our previous results in primary cells, here we have assessed oxidative stress levels in CD34+ hematopoietic progenitor cells (HSPCs) from myelofibrosis (MF) patients. In the present study, we describe how *JAK2* and *CALR* mutations impact on oxidative stress response in CD34+ cells. Finally, we measured total antioxidant capacity (TAC), an analyte frequently used to assess the antioxidant status of biological samples, in plasma of *JAK2* and *CALR*-mutated patients.

## 2. Materials and Methods

### 2.1. Patients and Samples

This study was conducted using Human CD34+ Hematopoietic Stem/Progenitor Cells (HSPCs) and plasma samples. Human CD34+ cells were purified from peripheral blood (PB) of 17 healthy donor (HD) and from 34 patients with a diagnosis of primary (*n* = 18) and secondary (*n* = 16) myelofibrosis (MF), 20 of them harboring *JAK2* mutations (i.e., *JAK2V617F* or *JAK2 exon 12* mutations) and 14 of them harboring *CALR* mutations (i.e., *type 1/type 1-like* or *type 2/type 2-like*). Plasma samples were collected from 129 MF patients (*n* = 86 *JAK2*, *n* = 43 *CALR*). All subjects, recruited from 4 Italian centers, provided informed written consent. MF was diagnosed according to 2016 World Health Organization criteria [26]. The study was conducted in accordance with the Declaration of Helsinki and was approved by local ethics committees.

### 2.2. Plasma Isolation

Blood samples were collected via venipuncture in EDTA-containing tubes. Plasma was separated as already described [27]. Hemolyzed samples were identified by spectrophotometric analyses, measuring the absorbance of hemoglobin at 414 nm using the NanoDrop ND-1000 spectrophotometer (Thermo Fisher Scientific, Waltham, MA, USA). As reported in literature [28], samples with a hemoglobin absorbance >0.3 were considered hemolyzed and were excluded from the study. Plasma was then aliquoted and stored at −80 °C.

### 2.3. Human CD34+ Hematopoietic Stem/Progenitor Cells Purification

Human CD34+ cells were collected from peripheral blood (PB) using a Ficoll-Hypaque density gradient. Briefly, blood sample was diluted 1:4 with Phosphate-Buffered Saline (PBS) with 2 mM EDTA and centrifuged at 800× *g* for 20 min at room temperature (RT) in a swinging-bucket rotor without brake. Following centrifugation, the mononuclear cell (MNC) ring was then recovered and washed twice with PBS. CD34+ were isolated through the immunomagnetic CD34+ positive selection using CD34 MicroBead Kit UltraPure (Miltenyi Biotec, Bergish Gladbach, Germany).

### 2.4. CD34+ Cells Culture Conditions

CD34+ cells were seeded in 24-well plates at 5 × 10^5^ cells/mL in Iscove’s modified Dulbecco’s medium (IMDM) (EuroClone, Pero, Milan, Italy) containing 10% Human Serum (Sigma-Aldrich, St. Louis, MO, USA), SCF (50 ng/mL), FLT3-ligand (50 ng/mL), TPO (20 ng/mL), IL-6 (10 ng/mL) and IL-3 (10 ng/mL) (all from Miltenyi Biotec, Bergish Gladbach, Germany), as previously described [29]. To induce oxidative stress, CD34+ cells were treated with Melittin, the main constituent and principal toxin of bee venom with powerful oxidant activity [30]. Briefly, CD34+ cells were seeded at 1 × 10^6^ cells/mL in IMDM medium supplemented with 10% Human Serum and Melittin 5 µg/mL (Sigma-Aldrich, St. Louis, MO, USA) for 6 and 24 h at 37 °C in a humidified atmosphere with 5% CO_2_.

### 2.5. Detection of Intracellular ROS Levels

The redox-sensitive fluorochrome 5-(and 6)-chloromethyl-2′,7′-dichlorodihydroflurescein diacetate dye (CM-H2DCFDA) (Invitrogen, Waltham, MA, USA) was used to measure the intracellular ROS. Briefly, CD34+ cells were treated with Melittin 5 µg/mL and intracellular ROS concentration was evaluated at 6 and 24 h. In total, 5 × 10^4^ treated/untreated CD34+ cells were loaded with 2 μM CM-H2DCFDA for 20 min at 37 °C. Before flow cytometry analysis, the cells were removed from loading buffer and incubated in growth medium for 30 min at 37 °C. Data acquisition and analysis were performed using a BD FACSCanto II (Becton Dickinson, BD, Franklin Lakes, NJ, USA). At least 100,000 events were detected for each sample to guarantee the statistical significance. Data were analyzed by FlowJo version 10.8 (Becton Dickinson, BD, Franklin Lakes, NJ, USA).

### 2.6. Annexin V/PI Staining

Apoptosis was evaluated by Annexin V assay (Annexin V-FITC Kit, Trevigen Inc, Minneapolis, MN, USA) following manufacturer protocol. Briefly, 5 × 10^5^ CD34+ cells from 5 *JAK2* and 5 *CALR*-mutated samples were washed with cold PBS and incubated in 100 μL Annexin V incubation reagent for 15 min at room temperature in the dark. After staining, cells were analyzed by using a BD FACSCanto II (BD Biosciences; San Jose, CA, USA). At least 10,000 events were counted for each sample to ensure statistical relevance.

### 2.7. Measurement of SOD Activity

SOD activity was measured by means of Superoxide Dismutase (SOD) Colorimetric Activity Kit (Invitrogen, Waltham, MA, USA) following manufacturer instruction. Briefly, CD34+ cells were treated for 24 h with Melittin 5 µg/mL. Then, 1 × 10^6^ CD34+ cells have been lysed in 250 µL of cold PBS with Halt™ Protease and Phosphatase Inhibitor (Thermo Fisher Scientific, Waltham, MA, USA) through 3 cycles of 10 s (Amplitude: 50%) using Branson digital sonicator model 450 (Branson, Brookfield, CT, USA). The protein concentration was determined by using Bradford Reagent (Sigma-Aldrich, St. Louis, MO, USA) and SOD activity were normalized based on total amount of protein were used to perform the assay.

### 2.8. Measurement of 8-OHdG Levels

8-hydroxy-2′-deoxy-guanosine (8-OHdG) is one of the major DNA oxidative modifications that can be generated by hydroxylation of the deoxyguanosine residues. Levels of 8-OHdG can be detected by enzyme-linked immunosorbent assay (ELISA). To this end, genomic DNA was extracted from CD34+ cells after treatment with Melittin 5 μg/mL for 24 h by means of DNeasy Blood and Tissue kit (Qiagen, Hilden, Germany). The same amount of genomic DNA of CD34+ cells (1.5 µg) was resuspended in 100 µL water and used for the detection of 8-OHdG level by means of the OxiSelectTM Oxidative DNA Damage ELISA Kit (Cell Biolabs, San Diego, CA, USA), following the manufacture’s instruction.

### 2.9. Measurement of TAC Levels

Total antioxidant capacity was measured by means of the Total Antioxidant Capacity Assay Kit (Colorimetric) (ABCAM, Cambridge, UK), following the manufacturer’s instruction. Briefly, plasma samples have been diluted 1:50 in PBS and was added an equal amount of protein mask, reagent supplied by the kit that allows the analysis of the small antioxidant molecules. The assay is based on antioxidant-based conversion of Cu^2+^ ion into Cu^+^, and detection of reduced Cu^+^ ion chelated with a colorimetric probe at 570 nm. Standard curve was prepared by using Trolox, a water-soluble tocopherol analogue, and antioxidant capacity was quantified as molar Trolox equivalents.

### 2.10. Measurement of L-Lactate Levels

Levels of plasmatic L-Lactate were measured by means of Lactate-Glo™ Assay (Promega, Madison, WI, USA) following manufacturer’s instructions. Plasma samples from 43 MF patients were diluted 1:100 in PBS. This assay couples the detection of lactate oxidation and NADH production with a bioluminescent NADH detection system. The luminescent signal is proportional to the amount of lactate in the sample and increases until all lactate is consumed, at which point a stable luminescent signal is achieved.

### 2.11. Statistical Analysis

Data were analyzed with GraphPad Prism8 version 8.4.0 (Graph Pad Software, San Diego, CA, USA); *p* value < 0.05 was considered significant. Comparisons between healthy donors and patients were reported with median with 95% CI (confidence interval) and analyzed with Mann–Whitney U test. Comparisons between treated and not treated cells were reported with median with 95% CI and analyzed with Wilcoxon matched-pairs signed rank test. The comparison between 6 h and 24 h treated cells was reported with a histogram with median with 95% CI and analyzed with Wilcoxon matched-pairs signed rank test. Correlation with categorical variables was assessed using Mann–Whitney U test or, for multiple comparisons, using Kruskal–Wallis test, while correlation with continuous variables was tested using simple linear regression. To compare the distribution of a variable in patients’ groups, the Chi-square test was used. All the clinical parameters were evaluated at the time of samples collection. Overall survival (OS) was calculated from the date of sample collection to the date of last follow-up or death occurrence; OS analyses were performed with the Kaplan–Meier method and the log-rank test was used to compare the curves. Multivariate analyses for OS were carried out by means of Cox proportional hazard regression and Wald test was used to compute *p* value using R version 3.4.1 (R Core Team 2021, Vienna, Austria).

## 3. Results

### 3.1. Intracellular ROS Quantification and Apoptosis Evaluation in CD34+ Cells from JAK2 or CALR-Mutated MF Patients

Intracellular ROS levels were assessed by flow cytometry in CD34+ cells from 20 *JAK2-* and 14 *CALR*-mutated MF patients compared with 17 HDs (Figure S1), before and after the induction of oxidative stress by Melittin treatment as previously described [30].

**Figure 1:**
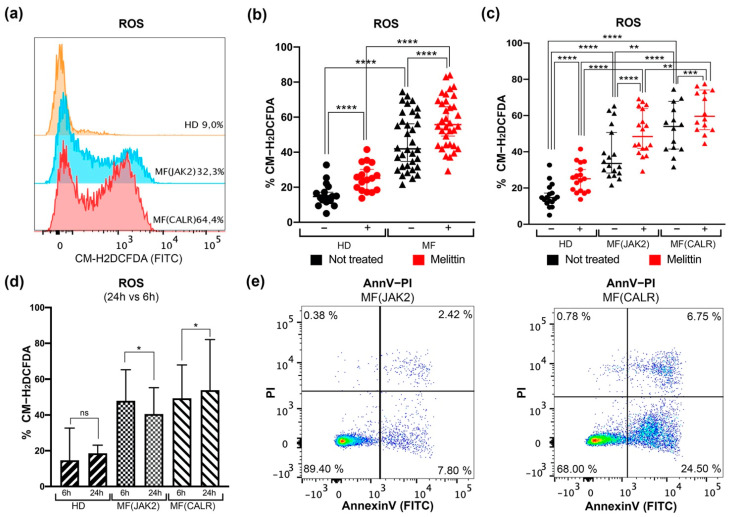
**Detection of intracellular ROS levels in CD34+ cells of MF patients.** (**a**) Representative histograms for flow cytometry detection of CM-H2DCFDA staining at 6 h in untreated CD34+ cells of HD, MF(*JAK2*) and MF(*CALR*) samples. (**b**) Dot plot shows the percentages of intracellular ROS assessed in HD and in MF samples, with (red dots) or without (black dots) 6 h of Melittin treatment respectively. The comparison between HD and MF was analyzed using Mann–Whitney U test, while the comparisons between treated and untreated cells were analyzed with Wilcoxon matched-pairs signed rank test. (**c**) Dot plot shows the percentages of intracellular ROS in the comparison between *JAK2-* or *CALR*-mutated MF patients compared to HD, with (red dots) or without (black dots) 6 h Melittin treatment respectively. The comparisons between HD and MF(*JAK2*) and between HD and MF(CALR) was analyzed with Mann–Whitney U test, while the comparisons between treated and not treated cells were analyzed with Wilcoxon matched-pairs signed rank test. (**d**) Histogram shows the percentages of intracellular ROS after 6 and 24 h; the statistical test used was Wilcoxon matched-pairs signed rank test. (**e**) Representative dot plots for flow cytometry detection of Annexin V/PI staining in CD34+ cells of MF(*JAK2*) and MF(*CALR*) samples. Data are reported as median with 95% CI (confidence interval). * *p* < 0.05, ** *p* < 0.01, *** *p* < 0.001, **** *p* < 0.0001.

Our data show that a statistically significant increase of ROS is measurable in MF untreated cells compared to HD cells (Figure 1a,b). Nevertheless, 6 h treatment with Melittin induces ROS accumulation in both MF and HD CD34+ cells (Figure 1b). Regarding *JAK2-* and *CALR*-mutated cells, Figure 1c shows that ROS production in CD34+ cells from *CALR*-mutated patients is significantly higher than in *JAK2*-mutated cells. Moreover, after Melittin treatment *CALR*-mutated cells show significantly higher ROS levels than *JAK2*-mutated ones (Figure 1c).

These differences are more remarkable after 24 h. *CALR*-mutated CD34+ cells are almost completely unable to reduce intracellular ROS level, while *JAK2*-mutated CD34+ cells are able to efficiently counteract the ROS accumulation, as evidenced by the lower percentage of ROS positive cells at 24 h of treatment (Figure 1d).

Finally, to investigate whether this different level of oxidative stress entails a different ability to induce cell death, apoptosis was evaluated by means of Annexin V/PI staining. Our results show an increase of apoptosis level in *CALR*-mutated CD34+ cells compared to *JAK2*-mutated ones, as shown in representative flow cytometry dot plots (Figure 1e).

These data further confirmed our previous results in *CALR*-mutated K562 cells [25], supporting a role for *CALR* mutation in ROS accumulation and in modulation of oxidative stress response in MF CD34+ primary cells. Moreover, our results showed that *CALR*-mutated samples, where we observed a greater increase of intracellular ROS, have a higher apoptosis level compared to *JAK2* ones.

### 3.2. Modulation of SOD Activity in CD34+ Cells from JAK2- or CALR-Mutated MF Patients

Among the response mechanisms to oxidative stress, SOD catalyzes the dismutation superoxide anions to hydrogen peroxide and oxygen, providing a defense against potential damage induced by ROS. To further study the cellular response to oxidative stress we assessed the SOD activity in wild-type and *JAK2-* or *CALR*-mutated CD34+ cells before and after 24 h Melittin treatment.

**Figure 2:**
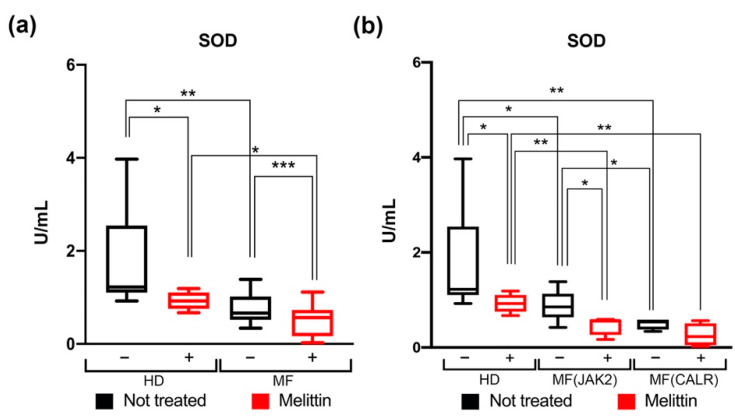
**Measurement of SOD activity in CD34+ cells of MF patients.** (**a**) Box plot shows the SOD activity in HD and in MF Scheme 24. hours Melittin treatment respectively. (**b**) Box plot shows the percentages of SOD activity in the comparison between *JAK2* or *CALR*-mutated MF patients compared to HD, with (red bars) or without (black bars) 24 h Melittin treatment respectively. SOD activity was normalized based on total amount protein were used to perform the assay. Data are reported as median of SOD activity (expressed in U/mL) with 95% CI (confidence interval). The comparisons between HD and MF were performed using Mann–Whitney U test, while the comparisons between treated and not treated cells were analyzed using Wilcoxon matched-pairs signed rank test. * *p* < 0.05, ** *p* < 0.01, *** *p* < 0.001.

Our data show that the SOD activity is significantly decreased in MF patients’ mutated cells compared to wild-type cells from HD in both Melittin treated and untreated condition (Figure 2a). Moreover, *CALR*-mutated cells show a greater decrease in SOD activity than *JAK2*-mutated ones (Figure 2b).

### 3.3. Quantification of Oxidative DNA Damage in CD34+ Cells from JAK2 and CALR-Mutated MF Patients

In order to study the DNA damage secondary to ROS accumulation [9], we measured 8-OHdG level, as a biomarker of oxidative stress-mediated DNA damage [31].

**Figure 3:**
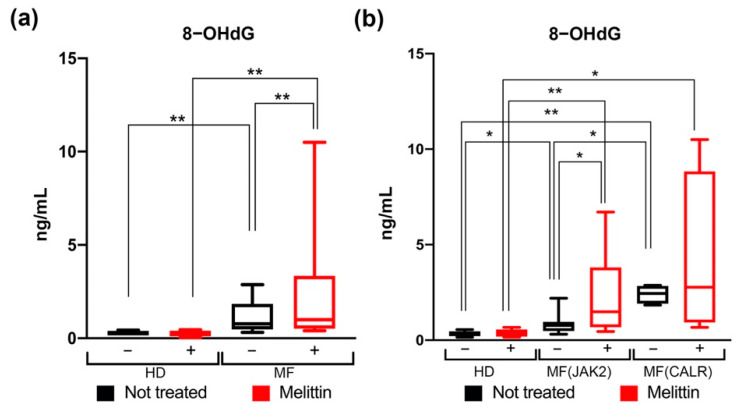
**Measurement of 8-OHdG levels in CD34+ cells of MF patients.** (**a**) Box plot shows the 8-OHdG levels in HD and in MF samples, with (red bars) or without (black bars) 24 h Melittin treatment respectively. (**b**) Box plot shows the levels of 8-OHdG in *JAK2-* or *CALR*-mutated MF patients compared to HD, with (red bars) or without (black bars) 24 h of Melittin treatment respectively. Data are reported as median of 8-OHdG levels (expressed in ng/mL) with 95% CI (confidence interval). The comparisons between HD and MF were analyzed by means of Mann–Whitney U test, while the comparisons between treated and not treated cells were analyzed using Wilcoxon matched-pairs signed rank test. * *p* < 0.05, ** *p* < 0.01.

Our results demonstrate that MF derived CD34+ cells have higher levels of 8-OHdG and this difference is even more evident after Melittin treatment, suggesting that, while cells from HDs are fully capable to respond to oxidative stress, mutated MF derived CD34+ cells display a greater sensitivity to oxidative DNA damage (Figure 3a).

Regarding the differences between *JAK2-* and *CALR*-mutated cells, once again our results suggest that the effect of oxidative stress on DNA damage is greater for *CALR*-mutated cells. *CALR*-mutated cells display higher 8-OHdG levels compared with HDs but also *JAK2*-mutated ones (Figure 3b).

### 3.4. Increased Plasma Levels of TAC in MF Patients Correlate with Clinical Detrimental Features

Based on the results obtained thus far on CD34+ cells showing increased DNA damage induced by enhanced ROS levels in MF patients. Suggesting that *CALR*-mutated cells appear to be more sensible to oxidative stress, it seemed appropriate to investigate whether, in MF patients with different driver mutations, we could identify a biomarker for the response to oxidative stress that can be used in a clinical setting.

TAC is defined as the moles of oxidants neutralized by one liter of body fluids, such as plasma [32,33,34]. TAC is a parameter suitable for routine use in order to investigate the oxidative stress in many pathological conditions; therefore, we evaluated the level of TAC in 129 plasma samples from MF patients harboring *CALR* or *JAK2* mutations (*n* = 86 *JAK2*, *n* = 43 *CALR*).

**Figure 4:**
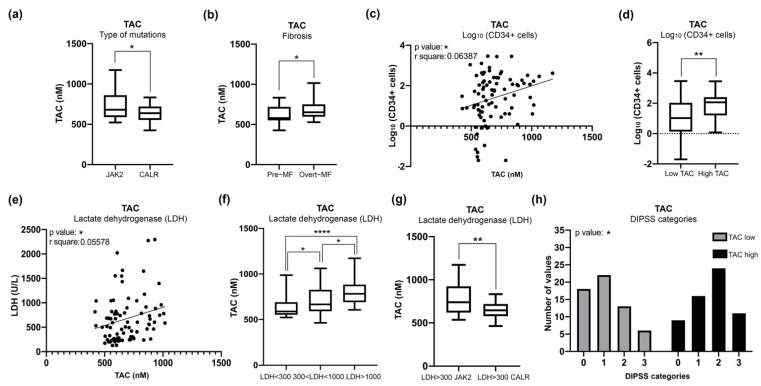
**Measurement of TAC in plasma of MF patients and correlation with clinical detrimental features.** (**a**) Box plot shows the level of TAC in *CALR* and *JAK2*-mutated MF patients. (**b**) Box plot shows correlation analysis of TAC level with fibrosis. (**c**) Graph represents linear regression analysis in MF patients showing correlation between the count of circulating CD34+ cells and TAC levels. (**d**) Box plot represents correlation analysis of the count of circulating CD34+ cells in MF samples presenting low or high levels of TAC. (**e**) Graph represents linear regression analysis in MF patients showing correlation between serum LDH and TAC levels. (**f**) Box plot represents correlation analysis of TAC levels with levels of serum LDH in MF samples divided into three ranges: LDH < 300, 300 < LDH < 1000, LDH > 1000 nM. (**g**) Graph showing correlations between *JAK2-* and *CALR*-mutated patients with LDH > 300 nM and TAC plasma levels. (**h**) Histogram was obtained from contingency tables computed to correlate low or high TAC plasma levels and DIPSS classification; the analysis was conducted with Chi-square test. Samples with low or high TAC levels are represented in gray and black, respectively. Box plot data are reported as median with 95% CI; the comparisons between two categories were analyzed with Mann–Whitney U test, while multiple comparisons were analyzed with Kruskal Wallis test. * *p* < 0.05, ** *p* < 0.01, **** *p* < 0.0001.

Figure 4a shows that *CALR*-mutated patients have significantly lower TAC level if compared to *JAK2*-mutated ones, suggesting that the *CALR* mutation somehow causes a reduced antioxidant response.

Next, in order to investigate the potential association between clinical features and TAC levels, we performed correlation analysis with a number of clinical parameters. Our results show that TAC activity significantly correlates with clinical parameters associated with MF severity, including the degree of fibrosis (Figure 4b), the numbers of circulating CD34+ cells (Figure 4c,d) and the levels of lactate dehydrogenase (LDH)(Figure 4e,f). Consistently with the data achieved thus far, among the patients with high levels of LDH, those who harbor *CALR* mutations display lower levels of TAC activity than those with *JAK2* mutations (Figure 4g).

Finally, we evaluated the correlation between TAC levels and patients’ prognostic classification. To this end patients were classified according to Dynamic International Prognostic Scoring System (DIPSS) [35] and patients’ cohort was stratified into two groups (Low and High) according to TAC plasma levels. The median value of TAC among MF samples (642,189 nM) was used as cutoff according to the “median split” method.

As shown in Figure 4h, the frequency of High TAC samples is increased in DIPSS Intermediate-2 and High classes while Low TAC activity is more common in DIPSS Low and Intermediate-1 categories. These data demonstrated that plasmatic TAC level is a detrimental feature that correlates with DIPSS classification.

### 3.5. JAK2-Mutant MF Patients with High Level of TAC Are Associated with a Poor OS

Given the described correlation between plasma TAC levels and detrimental clinical features we evaluated whether this parameter could be used as a marker of the patient’s ability to react to the oxidative injury and as an outcome predictor.

**Figure 5:**
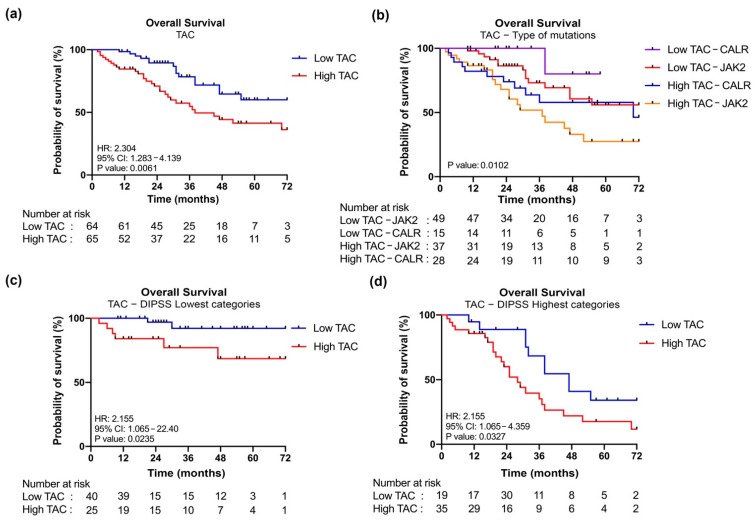
**Kaplan–Meier analysis of OS according to TAC plasma levels.** (**a**) Kaplan–Meier estimates OS according to TAC plasma levels. Patients’ cohort was stratified into two groups (low and high) according to the plasma levels of TAC. Blue and red curves represent patients with low or high levels of TAC respectively. (**b**) Kaplan–Meier estimates of OS according to TAC plasma levels and type of mutations. For this analysis patient cohort was stratified into four groups: *CALR*-mutated samples with low TAC (in violet), *JAK2*-mutated samples with low TAC (in red), *CALR*-mutated samples with high TAC (in blue), *JAK2*-mutated samples with high TAC (in yellow). (**c**) Kaplan–Meier estimates of OS according to TAC plasma levels in DIPSS lowest categories (Low and Intermediated-1). (**d**) Kaplan–Meier estimates of OS according to TAC plasma levels in DIPSS highest categories (High and Intermediated-2). Differences between two survival curves was evaluated by log-rank (Mantel–Cox) test. HR, hazard ratio computed to determine the magnitude of differences between two curves. 95% CI, 95% confidence interval. *p* value was computed by log-rank test.

Kaplan–Meier curves show that high levels of TAC correlate with a shorter OS (HR = 2.304, *p* = 0.0061) (Figure 5a). More specifically, patients could also be stratified according to driver mutation. When considering *JAK2*-mutated samples, high TAC levels identify a group of patients characterized by inferior OS, the same is true for *CALR*-mutated samples where this difference approaches the statistical significance (Figure 5b and Figure S2, Table 1).

Of particular interest, patients with *CALR* mutation and the lowest TAC activity are associated with better prognosis, while those with *JAK2* mutation and the highest levels of TAC activity are associated with worse outcome, as shown overall in Figure 5b and then by individual comparisons between the groups in Table 1.

Moreover, we performed a multivariate cox regression analysis demonstrating that High TAC classification represents a risk factor for inferior survival when considering both driver mutation and the other detrimental features (Table 2).

Finally, we performed survival analysis in samples dichotomized according to DIPSS to evaluate the independent prognostic value of TAC activity in MF. Patients with increased TAC activity display inferior survival when considering both DIPSS lowest (Low and Intermediate—1) and highest (Intermediate—2 and High) risk categories (Figure 5c,d) and multivariate analysis confirms that belonging to High TAC group is a risk factor for inferior survival independent from DIPSS classification (Hazard Ratio = 2.55; CI = 1.30–5.08; *p* = 0.006838).

## 4. Discussion

MF is a clonal stem cell disorder characterized by dysregulated proliferation of myeloid cells, extramedullary hematopoiesis and excessive production of pro-inflammatory cytokines, resulting in chronic inflammation and genomic instability [5,36]. About 90% of MF patients carry *JAK2*, *CALR* or *MPL* mutations, which are often mutually exclusive and are referred to as “driver” mutations [5,37].

In particular, *JAK2* is the most frequently mutated gene in MF and its mutation induces cytokine independence and constitutive activation of STAT proteins [13,38,39]. Moreover, it has been shown that *JAK2* mutation induces the accumulation of ROS in the hematopoietic stem cell compartment of knock-in (KI) mouse model and in myelofibrosis patients, suggesting the importance of oxidative stress in MF pathogenesis [16].

In 2013, somatic *CALR* mutations were identified in the majority of *JAK2* and *MPL* unmutated PMF and ET patients [17,18]. It has been shown that *CALR*-mutants binds to the Thrombopoietin receptor (TPO-R), causing its dimerization and leading again to the constitutive activation of JAK-STAT signaling pathway. Moreover, CALR overexpression has been shown to increase cell sensitivity to H_2_O_2_-induced cytotoxicity [24], indicating that CALR is also involved in the oxidative stress-induced apoptosis.

In the last decades, growing evidence has highlighted the role of oxidative stress in many tumors. Oxidative stress is considered as an imbalance between pro- and antioxidant species [1]. High levels of ROS are known to be involved in the pathogenesis of both solid and hematological cancers [40] and, in particular, a role in the initiation and progression of myeloproliferative disorders has been already described [2,3]. Here, we show that *JAK2* and *CALR* mutations impact differently on the oxidative stress in CD34+ cells from MF patients.

Our results demonstrate that MF CD34+ cells accumulate more ROS than those of healthy individuals, and ROS accumulation is associated with reduced SOD activity and increased DNA damage. These data show for the first time that the *CALR* mutation, more than *JAK2* one, is a condition favoring the increase of both oxidative stress and DNA damage-related genomic instability. Moreover, our results show that *CALR*-mutated CD34+ cells, which exhibit greater increase of intracellular ROS, have a higher apoptosis level compared to *JAK2*-mutated ones, suggesting that mutated-*CALR* patients could activate protective mechanisms promoting cell death, while a moderate increase of ROS induces a lower level of cell death and could permit the survival of cancer cells allowing disease progression.

Our previous data on the dysregulation of the oxidative response in K652 cells overexpressing mutated *CALR* support the hypothesis that the increase in oxidative stress that we have demonstrated here in *CALR*-mutated myelofibrosis stem cells depends precisely on a molecular pathway mediated by mutated *CALR* rather than on other disease related mechanisms [25]. However, further studies should be necessary to elucidate the mechanism by which *CALR* mutation is able to induce oxidative stress. In this regard, we previously demonstrated in *CALR*-mutated K562 cells the downregulation of OXR1, a sensor that plays a critical role in protecting the cell against oxidative stress [23,25]. Moreover, we can suppose that alterations in calcium levels induced by *CALR* mutations might affect its ability to interact with transcription factors regulating the response to oxidative stress [24]. We could speculate that *CALR* mutations might impact on the protein ability to interact with transcription factors regulating the response to oxidative stress.

As mentioned before, the final damage related to oxidative stress depends on the balance between the mechanisms supporting the production of ROS and the counterbalancing defense pathways. To further study this balance, we evaluated whether the differences in the level of oxidative stress between *JAK2* and *CALR*-mutated patients correspond to a different ability in reacting to this insult. The assessment of the TAC has already been widely used as an index of the cellular ability to respond to oxidative injury [41].

In our patients’ cohort, TAC levels significantly correlate with detrimental features such as the fibrosis grade, the peripheral CD34+ cells’ number and with the inflammatory response measured by LDH levels. Our data show that plasma samples from patients with MF harboring *CALR* mutations show lower TAC levels than patients harboring *JAK2* mutation.

In addition, since LDH is a critical enzyme of the anaerobic metabolic pathway, we also investigated the levels of L-Lactate in the plasma of MF patients. Cellular metabolism is strongly altered in pathological contexts and metabolic abnormalities is a hallmark of cancer. Lactate is produced by glycolysis, a major metabolic pathway responsible for glucose homeostasis and energy production [42]. Once considered merely a byproduct of glycolysis, lactate is now considered an important regulatory molecule of intermediate metabolism involved in cancer development and other diseases [43]. As shown in Figure S3, our results show an increase of L-Lactate in JAK2-mutated samples compared to *CALR* ones, suggesting that patients with the *JAK2* mutation have an increased anaerobic metabolism.

As a whole, these data suggest that *CALR*-mutated patients are not only exposed to greater oxidative injury, but also show a reduced capacity to respond to damage. Conversely, *JAK2*-mutated patients show higher TAC, LDH and L-Lactate levels than CALR mutated ones.

Therefore, finally, we evaluated the association between TAC plasma levels and OS. Survival analysis performed on the High and Low TAC stratified cohort, shows that low TAC values correlate with better outcome. A further stratification of patients also considering the driver mutation allowed us to observe that the levels of TAC are however the strongest variable in determining the outcome. Patients with highest plasmatic TAC levels show inferior survival within both *JAK2*-mutated and *CALR*-mutated cohorts, and patients with highest TAC levels harboring *JAK2* mutation display the worst prognosis, while patients with *CALR* mutations and low TAC levels show the best outcome. Moreover, multivariate analysis demonstrated that high TAC classification represents a prognostic factor for inferior survival independent from the type of driver mutation and the presence of other detrimental clinical features. The adverse prognostic impact of increased TAC activity on survival was also demonstrated by its correlation with DIPSS classification. Our results demonstrate that DIPSS highest categories are enriched in patients with high TAC levels, but we also observed that increased TAC is an independent prognostic factor allowing the identification of patients with inferior survival in both DIPSS lowest and highest categories.

In our working hypothesis, the biological interpretation of these results is to be sought in the balance between oxidative damage and response. A narrow concentration threshold determines whether ROS could act as second messengers, activating important cellular pathways, or induce cellular toxicity [44]. Moreover, ROS levels can be variable within cells, leading to an heterogeneity of induced damage [45]. Therefore, the type and degree of cellular response is crucial to determine the extent and intensity of ROS injury. High levels of ROS are able to induce oxidative stress, activating different cellular mechanisms which can lead to cell death. Conversely, a limited and moderate increase of ROS could permit the survival of DNA damaged cells [46]. As demonstrated by our results, *JAK2*-mutated CD34+ cells have lower levels of apoptosis and this might allow the survival of cancer cells, leading to the acquisition of new mutations that drive clonal evolution and disease progression, thus promoting the transition to the so-called “mutator-phenotype” described for MPNs [2,3].

This mechanistic hypothesis is also compatible with the already widely known evidence that patients with the *CALR* mutation generally have a better prognosis than patients with *JAK2-*mutation [47], and adds the evidence that the assessment of response to oxidative damage, showed here using an easy plasma assay, could provide an additional variable to better define the prognosis of patients with myelofibrosis.

## 5. Conclusions

In summary, our results confirmed that *CALR* mutation has a higher impact than *JAK2* mutation on the oxidative stress status in MF cells. Furthermore, *CALR*-mutated plasma samples have significantly lower TAC levels leading to a lower responsiveness to oxidative injury.

Increased TAC levels correlate with the presence of *JAK2* mutation and several detrimental clinical features. MF patients with high plasmatic TAC display inferior survival and multivariate analysis demonstrated that increased TAC activity might represent a novel prognostic biomarker independent from DIPSS classification and other detrimental features.

We speculated that the high increase in oxidative stress in mutated *CALR* patients could be involved in the activation of protective mechanisms which ultimately promote cell death, while in patients with the *JAK2* mutation, the slight increase in oxidative stress can be a mechanism that determines the persistence of cells with damaged DNA in which the accumulation of mutations promotes the disease progression.

## Figures and Tables

**Table 1:** **Individual comparisons between the four curves shown in Figure 5b**. Kaplan–Meier estimates of OS according to TAC plasma levels and type of mutations (Figure S2 shows individual extended data). Differences between two survival curves was evaluated by log-rank (Mantel–Cox) test. Hazard ratio was computed to determine the magnitude of difference between two curves. 95% CI, 95% confidence interval. Significant log-rank *p* values (*p* < 0.05) are represented in bold.

Comparison	Overall Survival	
	Hazard Ratio (95% CI)	*p*
**Low TAC–JAK2 vs. High TAC–JAK2**	2.487 (1.218–5.079)	**0.0068**
**Low TAC–CALR vs. High TAC–CALR**	5.240 (1.562–17.58)	0.0694
**Low TAC–JAK2 vs. Low TAC–CALR**	0.2756 (0.07882–0.9635)	0.1758
**High TAC–JAK2 vs. High TAC–CALR**	0.6290 (0.3073–1.288)	0.2132
**Low TAC–JAK2 vs. High TAC–CALR**	1.440 (0.6341–3.271)	0.3563
**Low TAC–CALR vs. High TAC–JAK2**	9.265 (3.642–23.57)	**0.0076**

**Table 2:** **Results of multivariate regression analysis for overall survival.** The prognostic impact of High TAC levels was determined while considering other detrimental features of MF patients by means of Cox regression analysis. Significant Wald test *p* values (*p* < 0.05) are represented in bold. 95% CI, 95% confidence interval.

Clinical Features	Overall Survival	
	Hazard Ratio (95% CI)	*p*
**Presence of JAK2V17F**	3.8620 (1.32–11.30)	**0.01365**
**Overt MF**	0.5023 (0.19–1.36)	0.17510
**Circulating CD34+ cells (Log10)**	1.7490 (1.05–2.90)	**0.03063**
**LDH > 1000 nM**	2.2210 (0.77–6.39)	0.13910
**High TAC classification**	2.8910 (1.18–7.1)	**0.006838**

## Data Availability

Data are contained within the article or Supplementary Materials.

## Supplementary Materials

The following are available online at https://www.mdpi.com/article/10.3390/antiox11010113/s1, Figure S1: Gating strategy for intracellular ROS detection by flow cytometry; Figure S2: Kaplan-Meier analysis of OS according to TAC plasma levels and type of mutations; Figure S3: Measurement of L-Lactate in MF patients’ plasma samples.

## References

[1] Sies H. (1986). Biochemistry of Oxidative Stress. Angew. Chem. Int. Ed. Engl..

[2] Bjørn M.E., Hasselbalch H.C. (2015). The Role of Reactive Oxygen Species in Myelofibrosis and Related Neoplasms. Mediat. Inflamm..

[3] Allegra A., Pioggia G., Tonacci A., Casciaro M., Musolino C., Gangemi S. (2020). Synergic Crosstalk between Inflammation, Oxidative Stress, and Genomic Alterations in BCR–ABL-Negative Myeloproliferative Neoplasm. Antioxidants.

[4] Tefferi A., Pardanani A. (2015). Myeloproliferative Neoplasms: A Contemporary Review. JAMA Oncol..

[5] Tefferi A. (2021). Primary Myelofibrosis: 2021 Update on Diagnosis, Risk--stratification and Management. Am. J. Hematol..

[6] Barosi G., Mesa R.A., Thiele J., Cervantes F., Campbell P.J., Verstovsek S., Dupriez B., Levine R.L., Passamonti F., On behalf of the International Working Group for Myelofibrosis Research and Treatment (IWG-MRT) (2008). Proposed Criteria for the Diagnosis of Post-Polycythemia Vera and Post-Essential Thrombocythemia Myelofibrosis: A Consensus Statement from the International Working Group for Myelofibrosis Research and Treatment. Leukemia.

[7] Vainchenker W., Kralovics R. (2017). Genetic Basis and Molecular Pathophysiology of Classical Myeloproliferative Neoplasms. Blood.

[8] Tefferi A., Vaidya R., Caramazza D., Finke C., Lasho T., Pardanani A. (2011). Circulating Interleukin (IL)-8, IL-2R, IL-12, and IL-15 Levels Are Independently Prognostic in Primary Myelofibrosis: A Comprehensive Cytokine Profiling Study. JCO.

[9] Yahata T., Takanashi T., Muguruma Y., Ibrahim A.A., Matsuzawa H., Uno T., Sheng Y., Onizuka M., Ito M., Kato S. (2011). Accumulation of Oxidative DNA Damage Restricts the Self-Renewal Capacity of Human Hematopoietic Stem Cells. Blood.

[10] Gloire G., Legrand-Poels S., Piette J. (2006). NF-ΚB Activation by Reactive Oxygen Species: Fifteen Years Later. Biochem. Pharmacol..

[11] Vener C., Novembrino C., Bamonti Catena F., Fracchiolla N.S., Gianelli U., Savi F., Radaelli F., Fermo E., Cortelezzi A., Lonati S. (2010). Oxidative Stress Is Increased in Primary and Post−polycythemia Vera Myelofibrosis. Exp. Hematol..

[12] Levine R.L., Wadleigh M., Cools J., Ebert B.L., Wernig G., Huntly B.J.P., Boggon T.J., Wlodarska I., Clark J.J., Moore S. (2005). Activating Mutation in the Tyrosine Kinase JAK2 in Polycythemia Vera, Essential Thrombocythemia, and Myeloid Metaplasia with Myelofibrosis. Cancer Cell.

[13] Kralovics R., Passamonti F., Buser A.S., Teo S.-S., Tiedt R., Passweg J.R., Tichelli A., Cazzola M., Skoda R.C. (2005). A Gain-of-Function Mutation of *JAK2* in Myeloproliferative Disorders. N. Engl. J. Med..

[14] Hurtado-Nedelec M., Csillag-Grange M.-J., Boussetta T., Belambri S.A., Fay M., Cassinat B., Gougerot-Pocidalo M.-A., Dang P.M.-C., El-Benna J. (2013). Increased Reactive Oxygen Species Production and P47phox Phosphorylation in Neutrophils from Myeloproliferative Disorders Patients with JAK2 (V617F) Mutation. Haematologica.

[15] Djikic D., Markovic D., Bogdanovic A., Mitrovic-Ajtic O., Suboticki T., Diklic M., Beleslin-Cokic B., Bjelica S., Kovacic M., P Cokic V. (2018). Oxidative and Nitrosative Stress in Myeloproliferative Neoplasms: The Impact on the AKT/MTOR Signaling Pathway. J. BUON.

[16] Marty C., Lacout C., Droin N., Le Couédic J.-P., Ribrag V., Solary E., Vainchenker W., Villeval J.-L., Plo I. (2013). A Role for Reactive Oxygen Species in JAK2V617F Myeloproliferative Neoplasm Progression. Leukemia.

[17] Nangalia J., Massie C.E., Baxter E.J., Nice F.L., Gundem G., Wedge D.C., Avezov E., Li J., Kollmann K., Kent D.G. (2013). Somatic CALR Mutations in Myeloproliferative Neoplasms with Nonmutated JAK2. N. Engl. J. Med..

[18] Klampfl T., Gisslinger H., Harutyunyan A.S., Nivarthi H., Rumi E., Milosevic J.D., Them N.C.C., Berg T., Gisslinger B., Pietra D. (2013). Somatic Mutations of Calreticulin in Myeloproliferative Neoplasms. N. Engl. J. Med..

[19] Michalak M., Corbett E.F., Mesaeli N., Nakamura K., Opas M. (1999). Calreticulin: One Protein, One Gene, Many Functions. Biochem. J..

[20] Elf S., Abdelfattah N.S., Chen E., Perales-Paton J., Rosen E.A., Ko A., Peisker F., Florescu N., Giannini S., Wolach O. (2016). Mutant Calreticulin Requires Both Its Mutant C-Terminus and the Thrombopoietin Receptor for Oncogenic Transformation. Cancer Discov..

[21] Araki M., Yang Y., Masubuchi N., Hironaka Y., Takei H., Morishita S., Mizukami Y., Kan S., Shirane S., Edahiro Y. (2016). Activation of the Thrombopoietin Receptor by Mutant Calreticulin in CALR-Mutant Myeloproliferative Neoplasms. Blood.

[22] Araki M., Yang Y., Imai M., Mizukami Y., Kihara Y., Sunami Y., Masubuchi N., Edahiro Y., Hironaka Y., Osaga S. (2019). Homomultimerization of Mutant Calreticulin Is a Prerequisite for MPL Binding and Activation. Leukemia.

[23] Zhang Y., Liu L., Jin L., Yi X., Dang E., Yang Y., Li C., Gao T. (2014). Oxidative Stress–Induced Calreticulin Expression and Translocation: New Insights into the Destruction of Melanocytes. J. Investig. Dermatol..

[24] Ihara Y., Urata Y., Goto S., Kondo T. (2006). Role of Calreticulin in the Sensitivity of Myocardiac H9c2 Cells to Oxidative Stress Caused by Hydrogen Peroxide. Am. J. Physiol. Cell Physiol..

[25] Salati S., Genovese E., Carretta C., Zini R., Bartalucci N., Prudente Z., Pennucci V., Ruberti S., Rossi C., Rontauroli S. (2019). Calreticulin Ins5 and Del52 Mutations Impair Unfolded Protein and Oxidative Stress Responses in K562 Cells Expressing CALR Mutants. Sci. Rep..

[26] Arber D.A., Orazi A., Hasserjian R., Thiele J., Borowitz M.J., Le Beau M.M., Bloomfield C.D., Cazzola M., Vardiman J.W. (2016). The 2016 Revision to the World Health Organization Classification of Myeloid Neoplasms and Acute Leukemia. Blood.

[27] Fantini S., Rontauroli S., Sartini S., Mirabile M., Bianchi E., Badii F., Maccaferri M., Guglielmelli P., Ottone T., Palmieri R. (2021). Increased Plasma Levels of LncRNAs LINC01268, GAS5 and MALAT1 Correlate with Negative Prognostic Factors in Myelofibrosis. Cancers.

[28] Shah J.S., Soon P.S., Marsh D.J. (2016). Comparison of Methodologies to Detect Low Levels of Hemolysis in Serum for Accurate Assessment of Serum MicroRNAs. PLoS ONE.

[29] Bianchi E., Ruberti S., Rontauroli S., Guglielmelli P., Salati S., Rossi C., Zini R., Tagliafico E., Vannucchi A., Manfredini R. (2017). Role of MiR-34a-5p in Hematopoietic Progenitor Cells Proliferation and Fate Decision: Novel Insights into the Pathogenesis of Primary Myelofibrosis. IJMS.

[30] Gajski G., Domijan A.-M., Žegura B., Štern A., Gerić M., Novak Jovanović I., Vrhovac I., Madunić J., Breljak D., Filipič M. (2016). Melittin Induced Cytogenetic Damage, Oxidative Stress and Changes in Gene Expression in Human Peripheral Blood Lymphocytes. Toxicon.

[31] Shibutani S., Takeshita M., Grollman A.P. (1991). Insertion of Specific Bases during DNA Synthesis Past the Oxidation-Damaged Base 8-OxodG. Nature.

[32] Serafini M., Villano D., Spera G., Pellegrini N. (2006). Redox Molecules and Cancer Prevention: The Importance of Understanding the Role of the Antioxidant Network. Nutr. Cancer.

[33] Lettieri-Barbato D., Tomei F., Sancini A., Morabito G., Serafini M. (2013). Effect of Plant Foods and Beverages on Plasma Non-Enzymatic Antioxidant Capacity in Human Subjects: A Meta-Analysis. Br. J. Nutr..

[34] Peluso I., Cavaliere A., Palmery M. (2016). Plasma Total Antioxidant Capacity and Peroxidation Biomarkers in Psoriasis. J. Biomed. Sci..

[35] Passamonti F., Cervantes F., Vannucchi A.M., Morra E., Rumi E., Pereira A., Guglielmelli P., Pungolino E., Caramella M., Maffioli M. (2010). A Dynamic Prognostic Model to Predict Survival in Primary Myelofibrosis: A Study by the IWG-MRT (International Working Group for Myeloproliferative Neoplasms Research and Treatment). Blood.

[36] Tefferi A. (2000). Myelofibrosis with Myeloid Metaplasia. N. Engl. J. Med..

[37] Pardanani A.D., Levine R.L., Lasho T., Pikman Y., Mesa R.A., Wadleigh M., Steensma D.P., Elliott M.A., Wolanskyj A.P., Hogan W.J. (2006). MPL515 Mutations in Myeloproliferative and Other Myeloid Disorders: A Study of 1182 Patients. Blood.

[38] James C., Ugo V., Le Couédic J.-P., Staerk J., Delhommeau F., Lacout C., Garçon L., Raslova H., Berger R., Bennaceur-Griscelli A. (2005). A Unique Clonal JAK2 Mutation Leading to Constitutive Signalling Causes Polycythaemia Vera. Nature.

[39] Levine R.L., Pardanani A., Tefferi A., Gilliland D.G. (2007). Role of JAK2 in the Pathogenesis and Therapy of Myeloproliferative Disorders. Nat. Rev. Cancer.

[40] Waris G., Ahsan H. (2006). Reactive Oxygen Species: Role in the Development of Cancer and Various Chronic Conditions. J. Carcinog..

[41] Bartosz G. (2003). Total Antioxidant Capacity. Advances in Clinical Chemistry.

[42] Philp A., Macdonald A.L., Watt P.W. (2005). Lactate—A Signal Coordinating Cell and Systemic Function. J. Exp. Biol..

[43] Brooks G.A. (2018). The Science and Translation of Lactate Shuttle Theory. Cell Metab..

[44] Lushchak V.I. (2014). Free Radicals, Reactive Oxygen Species, Oxidative Stress and Its Classification. Chem. Biol. Interact..

[45] Testa U., Labbaye C., Castelli G., Pelosi E. (2016). Oxidative Stress and Hypoxia in Normal and Leukemic Stem Cells. Exp. Hematol..

[46] Sabharwal S.S., Schumacker P.T. (2014). Mitochondrial ROS in Cancer: Initiators, Amplifiers or an Achilles’ Heel?. Nat. Rev. Cancer.

[47] Beer P.A., Campbell P.J., Green A.R. (2010). Comparison of Different Criteria for the Diagnosis of Primary Myelofibrosis Reveals Limited Clinical Utility for Measurement of Serum Lactate Dehydrogenase. Haematologica.

