# Supplementary material for: The Response to Oxidative Damage Correlates with Driver Mutations and Clinical Outcome in Patients with Myelofibrosis
Source: Antioxidants (Basel). 2022 Jan 5;11(1):113. doi: 10.3390/antiox11010113 (PMC8772737; doi:10.3390/antiox11010113)
Supplement: Supplementary file 1 [file antioxidants-11-00113-s001.zip › antioxidants-1516861-supplementary.pdf]

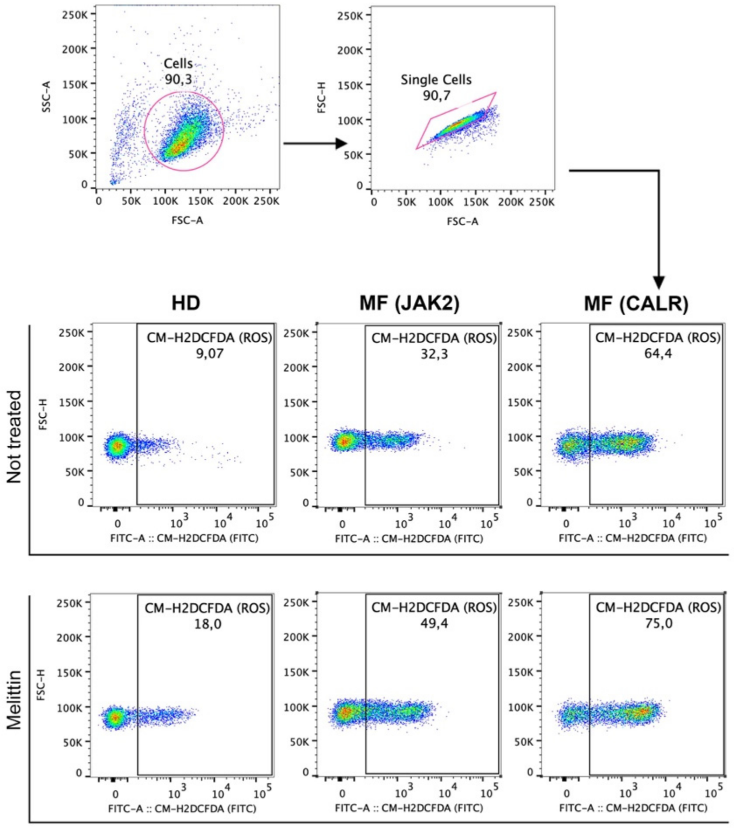

Figure S1. Gating strategy for intracellular ROS detection by flow cytometry. CD34+ cells were gated based on forward scatter (FSC) and side scatter (SSC) and doublets were excluded. Unstained controls for CD34+ cells were used to sharply define the CD34+ population positive to the 5-(and 6)-chloromethyl-2′,7′-dichlorodihydroflurescein diacetate dye (CM-H2DCFDA) compound. The data show intracellular ROS levels in Melittin treated/untreated samples after 6 hours in *JAK2* and *CALR*-mutated MF patients vs HD.

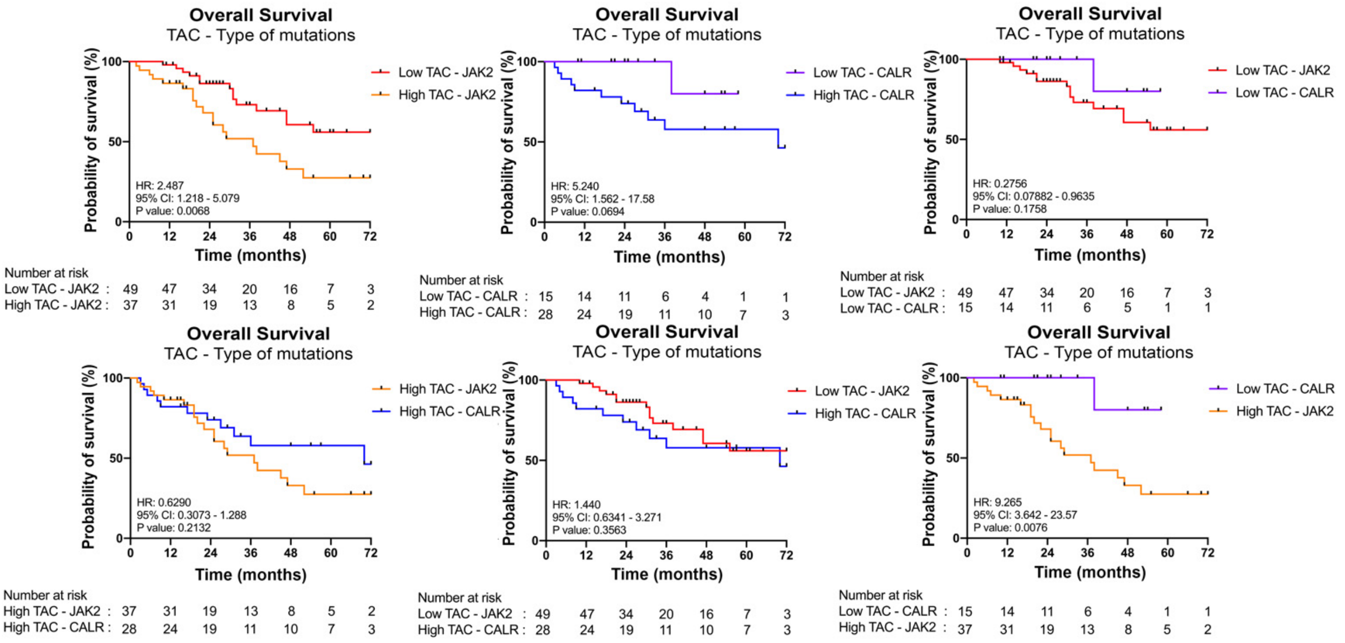

Figure S2. Kaplan-Meier analysis of OS according to TAC plasma levels and type of mutations. Patients' cohort was stratified into two groups (Low and High) according to the plasma levels of TAC. For this analysis patient cohort was further stratified into four groups: *CALR*-mutated samples with low TAC (in violet), *JAK2*-mutated samples with low TAC (in red), *CALR*-mutated samples with high TAC (in blue), *JAK2*-mutated samples with high TAC (in yellow). The curves shown here refer to individual comparisons between the four curves shown in Figure 5b. Differences between two survival curves was evaluated by Log-rank (Mantel-Cox) test. HR= hazard ratio computed to determine the magnitude of differences between two curves. 95% CI= 95% confidence interval. P-value was computed by log-rank test.

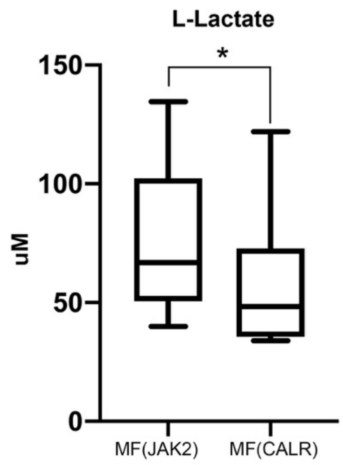

Figure S3. Measurement of L-Lactate in MF patients' plasma samples. Box plot shows the level of L-Lactate in *CALR* and *JAK2*-mutated MF patients. Data are reported as median L-Lactate concentration (expressed in uM) with 95% CI (CI= confidence interval). The comparisons between HD and MF were analyzed with Mann-Whitney U test. *p <0.05.
